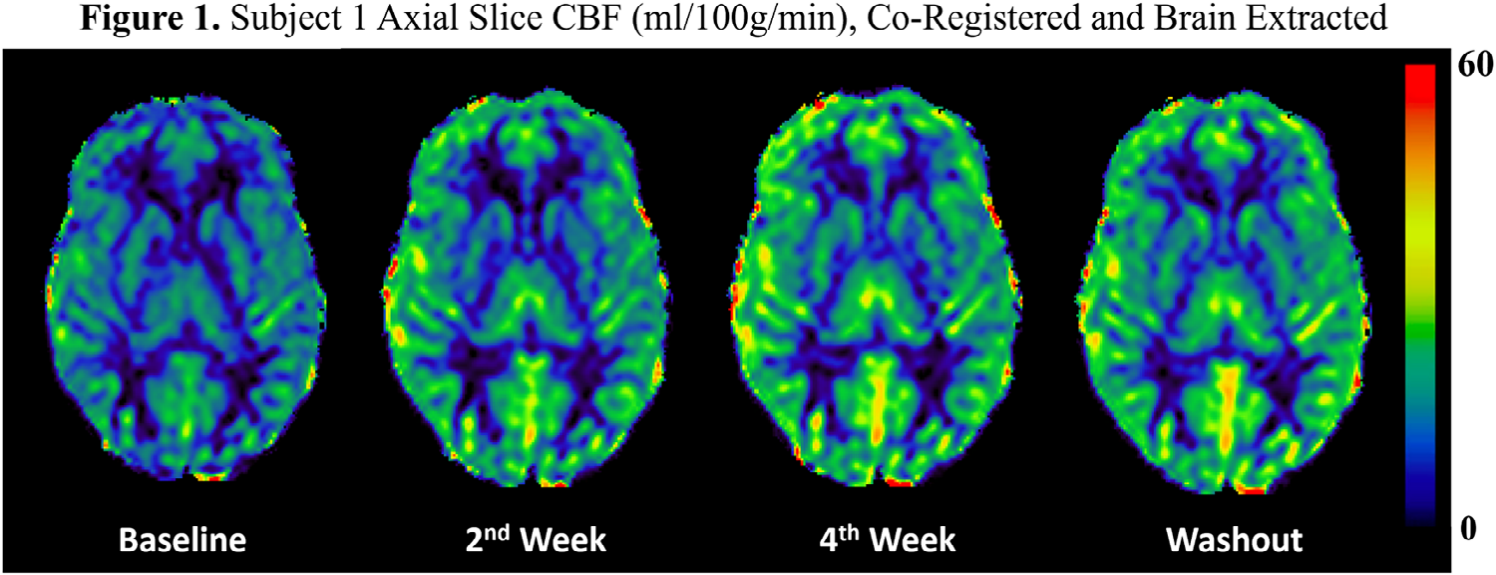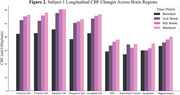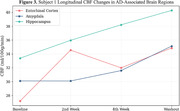# Short‐term Sirolimus Treatment Increases Cerebral Blood Flow in Asymptomatic APOE4 Females Determined by 3T MRI

**DOI:** 10.1002/alz.086993

**Published:** 2025-01-09

**Authors:** Caitlin M. Neher, Oleksandr Khegai, Jessica Overschmidt, Joanne Cassani, Xin Xing, Abeoseh Flemister, Stefan J. Green, Mehmet Kurt, David Q. Beversdorf, Feng Xu, John W. Grinstead, Priti Balchandani, Talissa A. Altes, Ai‐Ling Lin

**Affiliations:** ^1^ University of Washington, Seattle, WA USA; ^2^ Icahn School of Medicine at Mount Sinai Hospital, New York City, NY USA; ^3^ University of Missouri, Columbia, MO USA; ^4^ University of Kentucky, Lexington, KY USA; ^5^ Rush University, Chicago, IL USA; ^6^ Johns Hopkins University, Baltimore, MD USA; ^7^ Siemens Healthineers, Columbia, MO USA; ^8^ Icahn School of Medicine at Mount Sinai, New York, NY USA

## Abstract

**Background:**

Apolipoprotein ε4 allele (APOE4) is the strongest genetic risk factor for late‐onset Alzheimer’s disease (AD) with females having higher risk than males. Compared with non‐carriers, cognitively normal, middle‐aged APOE4 carriers have lower cerebral blood flow (CBF) decades before clinical symptoms appear. Early intervention to protect CBF would be critical for APOE4 carriers to mitigate AD progression. We have shown in APOE4 mice that Rapamycin (a.k.a. Sirolimus), a FDA‐approved mTOR‐inhibitor, can restore CBF. Here, in the first human study, our goal is to determine whether the findings translate to human APOE4 carriers by measuring CBF with MRI.

**Method:**

The study was performed at the University of Missouri. Low dose Sirolimus (1 mg/day) was given for 4 weeks to two middle‐aged, cognitively normal APOE4 females (45‐65 yrs; MOCA>28). APOE status was determined by oral swabs. 3T MRI‐based pseudo‐continuous arterial spin‐labeled (PCASL, 1.7x1.7x4mm resolution) images were acquired at four time‐points: baseline (pre‐treatment), 2nd and 4th week into treatment, and washout (2 weeks post‐treatment). PCASL data was processed with FSL BASIL toolbox and regionally analyzed using FreeSurfer segmentation of T1‐weighted images.

**Result:**

Average values of CBF (ml/100g/min) increased in all areas of investigation. When compared to baseline values, we observed an average 34.8±2.1% increase in washout CBF in the cortex across subjects (Figure 1). Within the cortex, there was an average increase of 41.5±8.9, 28.2±6.2, 37.4±1.7, and 33.1±0.3% in the frontal, parietal, temporal and occipital lobes, respectively. White matter CBF showed a similar trend, increasing by 33.7±4.9% across subjects from baseline to washout (Figure 2). Smaller AD‐associated regions were evaluated and also showed CBF improvement: the entorhinal cortex showed a 25.1±4.3% increase, while the hippocampus and amygdala showed a 21.4±1.1 and 23.6±9.8% increase, respectively (Figure 3). No side effects were observed, and no changes in blood glucose and HbAc1 levels were found in the participants.

**Conclusion:**

Short‐term Sirolimus treatment can effectively increase CBF for asymptomatic APOE4 females, who have the highest risk for AD. Future work will include APOE4 males, increase the sample size and compare CBF with non‐carriers (e.g., APOE3/APOE2). Restoration of CBF may pave a way to mitigate or prevent AD developments for APOE4 carriers.